# Transcriptomic and proteomic dynamics in the metabolism of a diazotrophic cyanobacterium, *Cyanothece* sp. PCC 7822 during a diurnal light–dark cycle

**DOI:** 10.1186/1471-2164-15-1185

**Published:** 2014-12-29

**Authors:** David Welkie, Xiaohui Zhang, Meng Lye Markillie, Ronald Taylor, Galya Orr, Jon Jacobs, Ketaki Bhide, Jyothi Thimmapuram, Marina Gritsenko, Hugh Mitchell, Richard D Smith, Louis A Sherman

**Affiliations:** Department of Biological Sciences, Purdue University, West Lafayette, IN USA; Environmental Molecular Sciences Laboratory, Pacific Northwest National Laboratory, Richland, WA USA; Biological Sciences Division, Pacific Northwest National Laboratory, Richland, WA USA; Bioinformatics Core, Purdue University, West Lafayette, IN USA

**Keywords:** *Cyanothece*, Cyanobacteria, RNA-Seq, N_2_ fixation, Proteomics, Butanol, CRISPR

## Abstract

**Background:**

*Cyanothece* sp. PCC 7822 is an excellent cyanobacterial model organism with great potential to be applied as a biocatalyst for the production of high value compounds. Like other unicellular diazotrophic cyanobacterial species, it has a tightly regulated metabolism synchronized to the light–dark cycle. Utilizing transcriptomic and proteomic methods, we quantified the relationships between transcription and translation underlying central and secondary metabolism in response to nitrogen free, 12 hour light and 12 hour dark conditions.

**Results:**

By combining mass-spectrometry based proteomics and RNA-sequencing transcriptomics, we quantitatively measured a total of 6766 mRNAs and 1322 proteins at four time points across a 24 hour light–dark cycle. Photosynthesis, nitrogen fixation, and carbon storage relevant genes were expressed during the preceding light or dark period, concurrent with measured nitrogenase activity in the late light period. We describe many instances of disparity in peak mRNA and protein abundances, and strong correlation of light dependent expression of both antisense and CRISPR-related gene expression. The proteins for nitrogenase and the pentose phosphate pathway were highest in the dark, whereas those for glycolysis and the TCA cycle were more prominent in the light. Interestingly, one copy of the *psbA* gene encoding the photosystem II (PSII) reaction center protein D1 (*psbA4*) was highly upregulated only in the dark. This protein likely cannot catalyze O_2_ evolution and so may be used by the cell to keep PSII intact during N_2_ fixation. The CRISPR elements were found exclusively at the ends of the large plasmid and we speculate that their presence is crucial to the maintenance of this plasmid.

**Conclusions:**

This investigation of parallel transcriptional and translational activity within *Cyanothece* sp. PCC 7822 provided quantitative information on expression levels of metabolic pathways relevant to engineering efforts. The identification of expression patterns for both mRNA and protein affords a basis for improving biofuel production in this strain and for further genetic manipulations. Expression analysis of the genes encoded on the 6 plasmids provided insight into the possible acquisition and maintenance of some of these extra-chromosomal elements.

**Electronic supplementary material:**

The online version of this article (doi:10.1186/1471-2164-15-1185) contains supplementary material, which is available to authorized users.

## Background

The interest in utilizing cyanobacterial systems for producing biofuels is increasing as our understanding of metabolism in these photosynthetic microorganisms improves. The ability to utilize carbon acquired from the atmosphere as feedstock along with energy provided by light to produce valuable chemicals and fuels (e.g. ethanol, butanol, hydrogen gas, and fatty acid methyl esters) is even more appealing in diazotrophic strains, since they can grow in the absence of combined nitrogen. Members of the unicellular genus *Cyanothece* have strong diurnal and circadian cycling of central metabolic processes and the development of a stable genetic manipulation system has been successful in the strain *Cyanothece* sp. PCC 7822 (herein *Cyanothece* 7822) [1, 2]. In general, members of *Cyanothece* have well-defined rhythms when grown under N_2_-fixing conditions, using light/dark or continuous light illumination [3]. During the light phase of growth, light energy is utilized in photosynthesis to secure and store CO_2_ in intracellular carbohydrate granules. During the dark period, photosynthesis shuts down and respiration utilizes the glucose stored in carbohydrate granules to produce energy and to consume intracellular oxygen. This enables the O_2_-sensitive nitrogenase to function properly during the dark period. The nitrogenase enzymes allow the cell to fix atmospheric nitrogen, a process which also releases high levels of hydrogen gas [4].

*Cyanothece* sp. ATCC 51142 (herein *Cyanothece* 51142) has been studied in great detail and was the first of these unicellular diazotrophs to be sequenced. The high coordination and diurnal oscillations of main metabolic processes such as respiration, photosynthesis, and nitrogen fixation allowed for keen insights into the robust metabolisms of this strain. These characteristics led to a series of transcriptional studies [5–8] and improvements in hydrogen production [4, 9]. With the advancement of technology, proteomic studies were performed which unveiled a network of interacting protein systems that play off of each other, one system setting up the cellular environment for the next [10–15]. The work described in Aryal et al. [10] was the first to involve proteomics in *Cyanothece* 51142, in addition to transcriptomics, and demonstrated that many fewer proteins cycled than did transcripts. That study, and a proteomics comparison between *Cyanothece* 51142 and *Cyanothece* 7822 [14], indicated that both strains have a higher level of the nitrogenase, respiration and glycogen degradation enzymes in the dark than in the light. Thus, protein cycling throughout the day and night is of functional significance.

Research into *Cyanothece* 51142 continues, but mutant generation is problematic due to high levels of illegitimate recombination that prevents stable homologous recombination efforts. Due to this, the utility of *Cyanothece* 51142 as a biological chassis is limited. Min and Sherman [1] overcame this hurdle in *Cyanothece* 7822 with the first demonstration of stable genetic modification. This study showcased the difficulty of obtaining stable mutants in most *Cyanothece* strains and how to improve transformation efficiency by introducing linear, single-stranded, oligonucleotides carrying the desired mutations flanked by fragments homologous to a chromosomal insertion site. More recently, Zhang et al. [2] provided another example of stable genetic modification in *Cyanothece* 7822 with the generation of a *hupL* mutant. This mutant led to the unexpected finding that inactivating the uptake hydrogenase in *Cyanothece* 7822 lowered H_2_ evolution and nitrogenase activity, thus demonstrating the importance of HupLS in protecting nitrogenase from O_2_ toxicity.

Transcriptional and proteomic studies on *Cyanothece* 7822 thus far have compared the metabolic capabilities of *Cyanothece* 7822 with the other *Cyanothece* genus members. Recent work by Aryal et al. [14] reported the protein and transcript abundances in *Cyanothece* 51142 and 7822 under 8 conditions, including nitrogen depletion and mixotrophy. Another study has compared the proteomic expression of five *Cyanothece* strains in order to determine the metabolic capabilities within the *Cyanothece* strains [13]. These initial studies have shown that, although the two *Cyanothece* strains contain quite similar proteomes, they exhibit different protein abundances. These differences suggest the presence of varied cellular strategies in response to altered environmental conditions.

In this study, we utilized transcriptomic and proteomic methods to quantify the relationship between transcription and translation underlying central and secondary metabolism in response to nitrogen fixation at four time points across a 24 h period consisting of 12 hour light and 12 hour dark. We identified dynamic relationships between transcription and translation, suggesting that there are many levels of regulation in addition to that at the transcriptional level. Our results provide a better understanding of metabolism of *Cyanothece* 7822 in light and dark conditions and how the metabolic partitioning can be of importance for biofuel production.

## Results

### RNA-Sequencing and microarray correlation

*Cyanothece* 7822 was grown in nitrogen-free BG-11 media [16] with reduced phosphate [17] under 12 h light – 12 h dark conditions for three consecutive days. On the third day of exponential growth, cells were collected for RNA-sequencing (RNA-seq) and proteomics at four time points across the light/dark cycle. Material was collected at the beginning of the light period (L0/D12), three hours into the light period (L3), at the end of the light period (L12/D0), and three hours into the dark period (D3). These time points represent sunrise (L0), mid-morning (L3), sunset (D0) and mid-night (D3) and display the full range of expression differences as previously demonstrated in *Cyanothece* 51142 [5, 7, 8].

**Figure 1:**
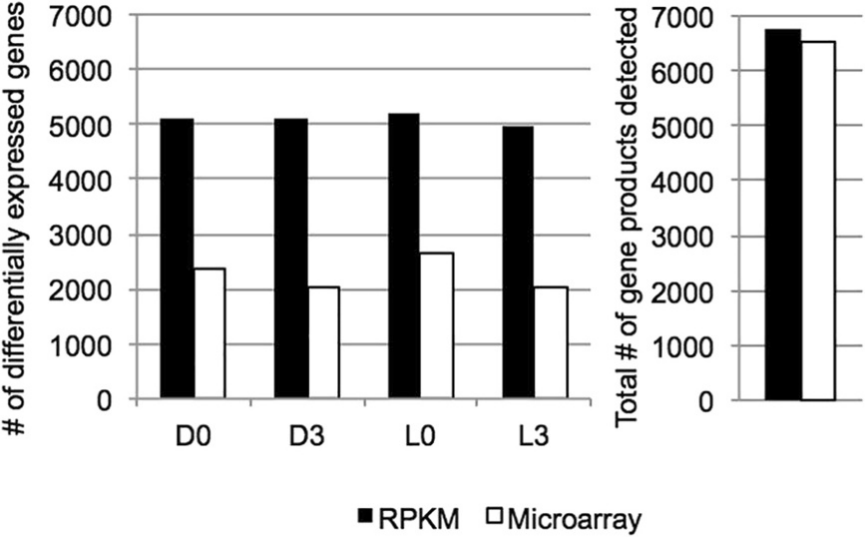
**Comparison of gene expression at 4 time periods in** ***Cyanothece*** **7822 grown under N**_**2**_ **-fixing conditions in 12 h light-12 h dark measured through RNA-sequencing (RPKM) and by microarray.**

Since this represented the first RNA-seq analysis for *Cyanothece* 7822 mRNA transcripts, we compared the results with those from a microarray platform that was constructed as described previously for *Cyanothece* 51142 and could provide a frame of reference for the results [7, 8]. Transcriptional expression data was consistent between the two methods. Overall, 6766 and 6539 gene products were detected in total from the RNA-seq and microarray, respectively, and there were ~2-fold more differentially expressed (DE) genes detected in the RNA-seq at all time points measured (Figure 1 and Additional file 1: Table S1 and Additional file 2: Table S2). This difference was as expected, as similar comparisons have shown that RNA-seq has a lower background signal and a larger dynamic range in expression levels compared to microarrays [18–20].

Statistical comparison indicated that results obtained from the two platforms were highly comparable. Scatter plots were generated using log_2_ fold change (FC) values of significantly differentially expressed genes (adj-p ≤ 0.05) from D0 vs. L0, D3 vs. L0 and L3 vs. L0 (Additional file 3: Figure S1) and the Spearman correlation coefficients for each of the comparisons used to generate the scatter plots were calculated using ‘cor’ function in R. These coefficient values are listed in Additional file 1: Table S1. Spearman values, especially for significant genes, are comparable with similar studies [18]. With this as a basis, our analysis focused on the RNA-seq data as it provided a more complete analysis of the differential transcription. Additional file 4: Figure S2 is a comparison heat map showing the expression patterns of genes from the main functional categories.

### Light-dependent expression of functionally relevant pathways

The unicellular diazotrophs of the genus *Cyanothece* follow strong light–dark oscillations for the major physiological functions of nitrogen fixation, respiration and photosynthesis. Thus, we chose sample times that would enable us to determine the type of transcriptional oscillations in *Cyanothece* 7822 and allow us to compare our results to that of *Cyanothece* 51142. This permitted us to discuss differential transcription between early (L3 vs. L0 and D3 vs. D0, and late (D0 [L12] vs. L3, and L0 [D12] vs. D3) light and dark periods, respectively.

**Figure 2:**
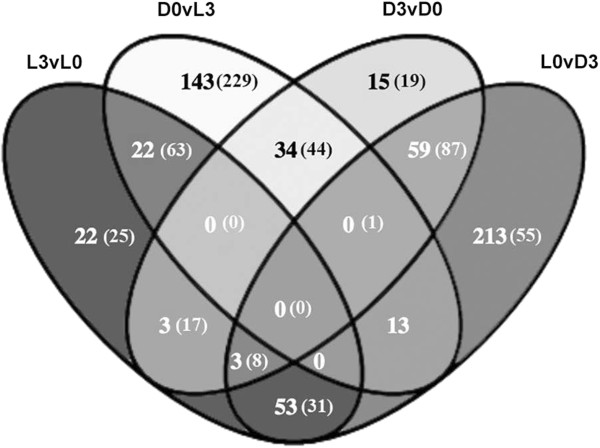
**Venn diagram displaying the number of genes significantly up (bold lettering) and down (in parentheses) regulated ≥2-fold across the various time points measured.** Detailed listing of each group can be found in Additional file 5: Table S3.

In Figure 2, the number of genes significantly (adj-p < 0.05) upregulated 2-fold (>2 FC) and downregulated 2-fold (<0.5 FC) are presented. Prior to the transition into the light and dark periods (D0 and L0, respectively), we see the highest number of differentially expressed genes consistent with the understanding that the cell needs to strongly adjust its enzymatic repertoire to meet the different needs of photosynthesis during the light period, and glycolytic processes and nitrogen fixation during the dark. Observing the expression of core metabolic genes peaking at the onset (e.g., L1 and D1 for *Cyanothece* 51142) or in the case of *Cyanothece* 7822 in this study, L0 and D0, is a notable characteristic of diurnal gene regulation in cyanobacteria [7]. At D0 (L12) vs. L3, we see the expression of genes for dark metabolic activities such as hydrogenase, nitrogenase, and TCA cycle. At L0 (D12) vs. D3, genes for light metabolism, such as photosynthesis and carbohydrate storage, begin to be upregulated as the cell enters the light phase. Details of each comparison population are provided in Additional file 5: Table S3. This table has a worksheet for each of the comparisons shown in the Venn diagram, identifying each gene and (gene function) that is differentially regulated.

**Figure 3:**
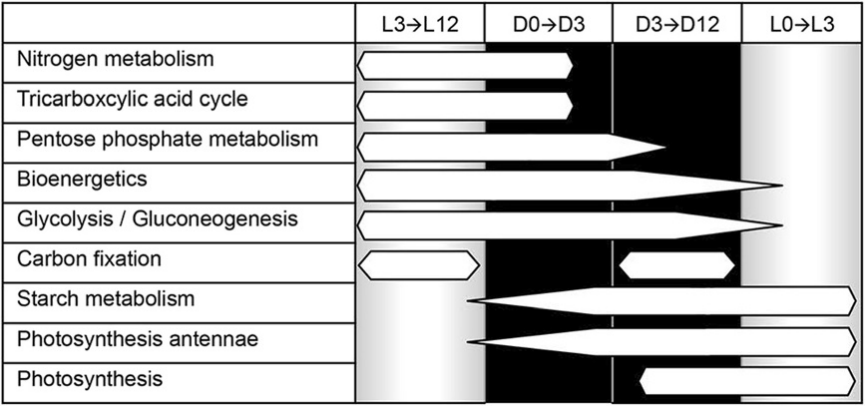
**Diagram of differential gene expression in** ***Cyanothece*** **7822 grown in 12-h light 12-h dark. Bar thickness is a qualitative measure of the mRNA levels at that time point.** The two dark segments (early and late dark) are highlighted in black. The figure begins with the late light period (D0 vs L3) on the left to best represent the pattern of gene expression throughout the day to the early light period (L3 vs L0).

We concentrated our detailed analysis on the approximately 500 genes that comprise the major metabolic processes required for growth under diazotrophic, light–dark conditions, although the data for all genes is presented in the supplemental tables. During the light period, we observe high expression of genes related to bioenergetics, such as various NADH-quinone oxidoreductases and NAD(P)H dehydrogenases, and CO_2_ fixation genes, such as carbon dioxide concentrating mechanism genes, ribulose-bisphosphate carboxylase and carbonic anhydrase. Other light-enhanced genes are those involved in the gluconeogenesis pathway, as well as the reductive pentose phosphate pathway as indicated by the presence of ribulose-phosphate 3-epimerase. Most predominant are genes responsible for encoding proteins involved in photosynthesis and glycogen biosynthesis. As the cell enters the dark phase, many of these genes become down regulated and genes involved in nitrogen fixation and butanoate metabolism (polyhydroxyalkonate synthesis, short-chain dehydrogenase, and acetyl-CoA acetyltransferase) become upregulated. In addition, genes of the TCA cycle are highly expressed and genes encoding proteins in aerobic respiration, such as cytochrome oxidases, Rieske (2Fe-2S) domain-containing proteins, and the uptake and bi-directional hydrogenase were strongly upregulated. Of course, these proteins are also needed to deplete the intracellular oxygen levels in the cytoplasm in order to protect the oxygen-sensitive nitrogenase enzymes. Much of the key metabolic information contained in Additional file 1: Table S1 and Additional file 2: Table S2 and described above is visualized readily in Figure 3.

Interestingly, the analysis indicated that the initiation of transcription of genes encoding some key metabolic functions was not restricted to the light or to the dark exclusively, as in *Cyanothece* 51142 [8, 21]. For example, transcription of the N_2_-fixing enzymes, Cyan7822_3667 to 3684, was upregulated in both the late light and the early dark. Similarly, many enzymes involved in heme and pigment biosynthesis and genes encoding phycobiliproteins were upregulated >2FC at D3vsD0 and L0vsD3, indicating that expression was high throughout much of the dark period from three hours into the dark up to the end of the dark period. The genes for cytochrome oxidase and those involved in hydrogenase and uptake hydrogenase activity were transcribed in the light so that the function would be available once the nitrogenase proteins were available in the late light period (See Additional file 5: Table S3 and Additional file 6: Figure S3). In these cases, the genes were transcribed in preparation for the next important metabolic phase. All of these results are consistent with the physiological measurements [17], which demonstrated nitrogen fixation and hydrogen production just prior to the dark period.

Figure 3 emphasizes a few temporal points about transcription in *Cyanothece* 7822. First, transcription of important metabolic functions typically began in a light period prior to their maximum expression. Thus, nitrogen fixation genes transcription began in the light, but peaked at D3. The same was true for the TCA and much of the PPP cycle genes, as well as those involved with respiration. On the other hand, carbon fixation, starch metabolism and photosynthesis gene transcription began in the dark and peaked in the light. Therefore, the transcription was not as tightly synchronized as in *Cyanothece* 51142 (Figure 3).

**Figure 4:**
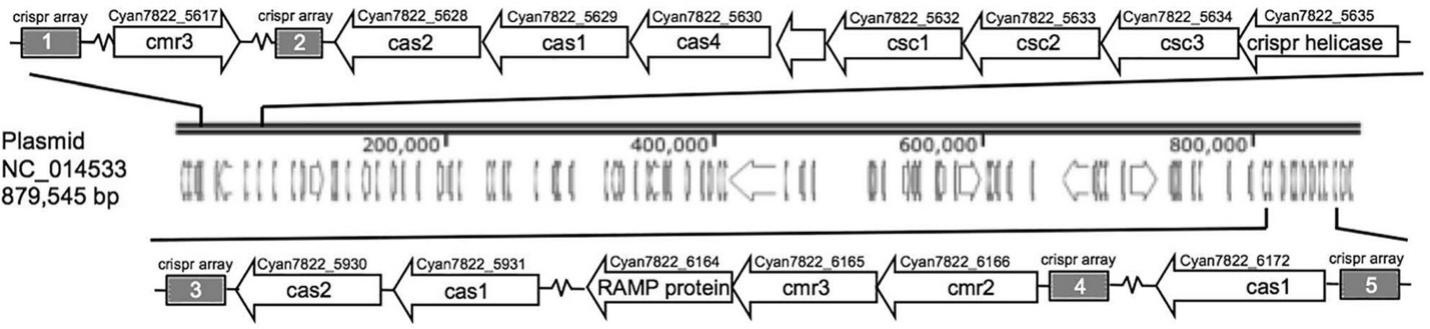
**CRISPR genes on the large, linear plasmid (NC_014533) are located on both ends of the fragment and are highly transcribed in the late dark, early light period.** Five repeat arrays were identified using CRISPRFinder and the CRISPRdb [22, 23] and are represented as bars with the numbers 1–5.

Unexpectedly, there was an upregulation of a Cmr4 family CRISPR-associated RAMP gene (*Cyan7822_6164*). Upon further investigation, a significant number of CRISPR (**c**lustered **r**egulatory **i**nterspaced **s**hort **p**alindromic **r**epeats) associated genes were expressed in *Cyanothece* 7822 and were mainly located near either end of the large linear plasmid (Figure 4). We will discuss the CRISPR genes in greater detail in the anti-sense and CRISPR expression section.

### Protein expression

One objective of this study was to compare and contrast protein expression with the transcriptional data in order to investigate in-phase and anti-phase abundance maxima [24]. We were able to generate a large protein dataset containing 1322 quantifiable proteins from the four time points. We again focused on proteins that were involved with key metabolic pathways as outlined in Additional file 2: Table S2. These proteins and their kinetics over the 4 time periods are shown in Additional file 7: Table S4. The experiment identified key proteins in each of the major functional categories that led to some important insights into the regulation of metabolism in this unicellular diazotroph.

Proteins involved with cellular bioenergetics were mainly present in the dark. The highest levels of the cytochrome oxidase operon proteins, Cyan7822_4711-4713, were found in the dark, suggesting that this complex was used for respiration during the N_2_-fixing dark phase. Only one protein from the other complex, Cyan7822_4378, was identified and it was more constant throughout the experiment, although it dropped to the lowest levels at L3. The NADH dehydrogenase subunit 1, Cyan7822_2520, had levels some 2X higher at D3 than at the other times, also indicating that this protein was part of a dark-inducible complex.

As also seen in *Cyanothece* 51142, proteins encoded by the nitrogenase cluster genes were differentially expressed with a peak at D3 for all proteins except for NifD, which peaked at the end of the dark period. In a complementary fashion, the proteins involved in glutamate metabolism tended to be at higher levels in the late dark to early light.

**Figure 5:**
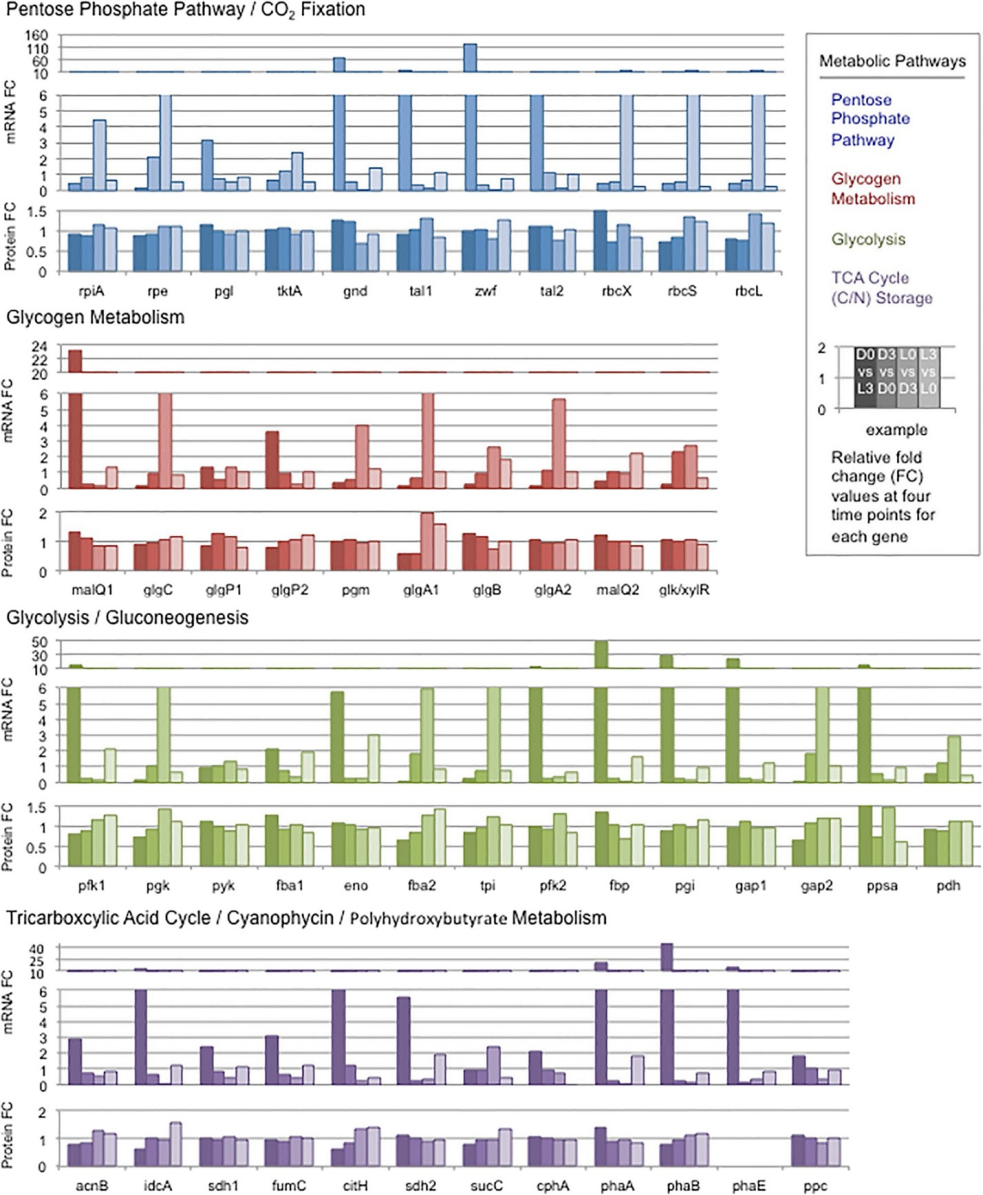
**The relative fold change expression levels of both mRNA and protein for 47 genes involved with central metabolism in** ***Cyanothece*** **7822 are shown.** The y-axis represents the relative fold change in mRNA level or the relative fold change in protein level. The x-axis represents the four comparisons: D0 vs. L3 (mid-day to sunset); D3 vs. D0 (mid-night to sunset); D3 vs. L0 (mid-night to sunrise); and, L3 vs. L0 (sunrise to early morning). The figure is colored coded as follows for the protein-mRNA expression dynamics. PPP, blue; glycogen metabolism, red; glycolysis, green; and TCA, purple. Genes within each groups displayed are as follows: **PPP and Carbon Fixation:** Cyan7822_0018, rpiA; Cyan7822_0124, rpe; Cyan7822_0172, pgl; Cyan7822_1076, tktA; Cyan7822_3017, gnd; Cyan7822_4172, tal1; Cyan7822_4173, zwf; Cyan7822_5424, tal2; Cyan7822_2900, rbcX; Cyan7822_2901, rbcS; Cyan7822_2899, rbcL. **Glycogen Metabolism:** Cyan7822_1182, *malQ1*; Cyan7822_2049, *glgC*; Cyan7822_2294, *glgP2*; Cyan7822_2322, *glgP1*; Cyan7822_2491, *pgm*; Cyan7822_2570, *glgA1*; Cyan7822_2889, *glgB*; Cyan7822_4734, *glgA2*; Cyan7822_5145, *malQ2*; Cyan7822_5605, *glk/xylR.* **Glycolysis:** Cyan7822_0159, *pfk1*; Cyan7822_1455, *pgk*; Cyan7822_1523, *pyk*; Cyan7822_1612, *fba1*; Cyan7822_1633, *eno*; Cyan7822_1659, *fba2* Cyan7822_1688, *tpi*; Cyan7822_3033, *pfk2*; Cyan7822_4171, *fbp*; Cyan7822_4412, *pgi*; Cyan7822_4423, *gap1*; Cyan7822_4638, *gap2*; Cyan7822_1627, *ppsa*; Cyan7822_2730, *pdh*. **TCA and C/N Storage:** Cyan7822_0752, *acnB*; Cyan7822_1400, *idcA*; Cyan7822_1551, *sdh1*; Cyan7822_2824, *fumC*; Cyan7822_3004, *citH*; Cyan7822_3071, *sdh2*; Cyan7822_3782, *sucC*; Cyan7822_2816, *cphB*; Cyan7822_1326, *phaA*; Cyan7822_1327, *phaB*; Cyan7822_1330, *phaE*; Cyan7822_2645, *pcc*.

Proteins involved in central metabolism (glycolysis, TCA cycle and PPP) demonstrated varied expression patterns (Figure 5). The figure displays the relative fold change of mRNA levels with time in the upper two panels for each metabolic pathway along with the fold change in proteins levels with respect to time in the lower panel. The figure demonstrates that protein levels typically did not vary more than 25% throughout the daily cycle. However, in specific cases, the expression patterns demonstrated a light vs. dark periodicity. For example, most of the proteins for glycolysis were similar in the light and in the dark. Nonetheless, both copies of phosphofructokinase (Pfk) were expressed; one peaked in the light (Cyan7822_0159) and one peaked in the late dark period (Cyan7822_3033). Similarly, two Fba proteins were expressed, one in the light (Cyan7822_1659) and one in the dark (Cyan7822_1612). Neither protein was found exclusively in one time period, but the data suggested that Fba1 (Cyan7822_1612) was strongly upregulated in the late dark and early light, whereas Fba2 (Cyan7822_1659) was upregulated more in the late light and early dark periods. In both cases, the proteins were at reasonable levels throughout the light and the dark, although the results suggested that different Pfk and Fba proteins were expressed at different times for slightly different cellular needs. The TCA cycle proteins that were identified were slightly higher in the light, indicating that this pathway is mostly involved with generating metabolites. Some of the proteins in the Pentose Phosphate Pathway, such as Rpi and Rpe, are also involved with CO_2_ fixation, and they were upregulated in the light. This paralled the induction of RuBP carboxylase, RbcLS, that was upregulated nearly 2-fold in the late-dark and early-light periods (Figure 5). On the other hand, PPP proteins were slightly upregulated in the early dark, and lowest in the late dark period, which suggested that a primary function was to provide reducing equivalents for N_2_ fixation during the early dark period. Importantly, glycogen synthase was strongly light regulated, appropriate for construction of the glycogen granules during carbon fixation. The two glycogen phosphorylase genes were both expressed as protein at all 4 time periods, but with slightly different kinetics: GlgP1 (Cyan7822_2322) had the highest abundance in the early light (L3), whereas GlgP2 (Cyan7822_2294) was highest in the late dark (L0). In a similar fashion, GlgA1 had a 3-fold higher level in the late-dark/early light than at other times, whereas GlgA2 was much more constant throughout the cycle.

### Relationship between transcription and translation

#### Nitrogen fixation and photosynthesis

**Figure 6:**
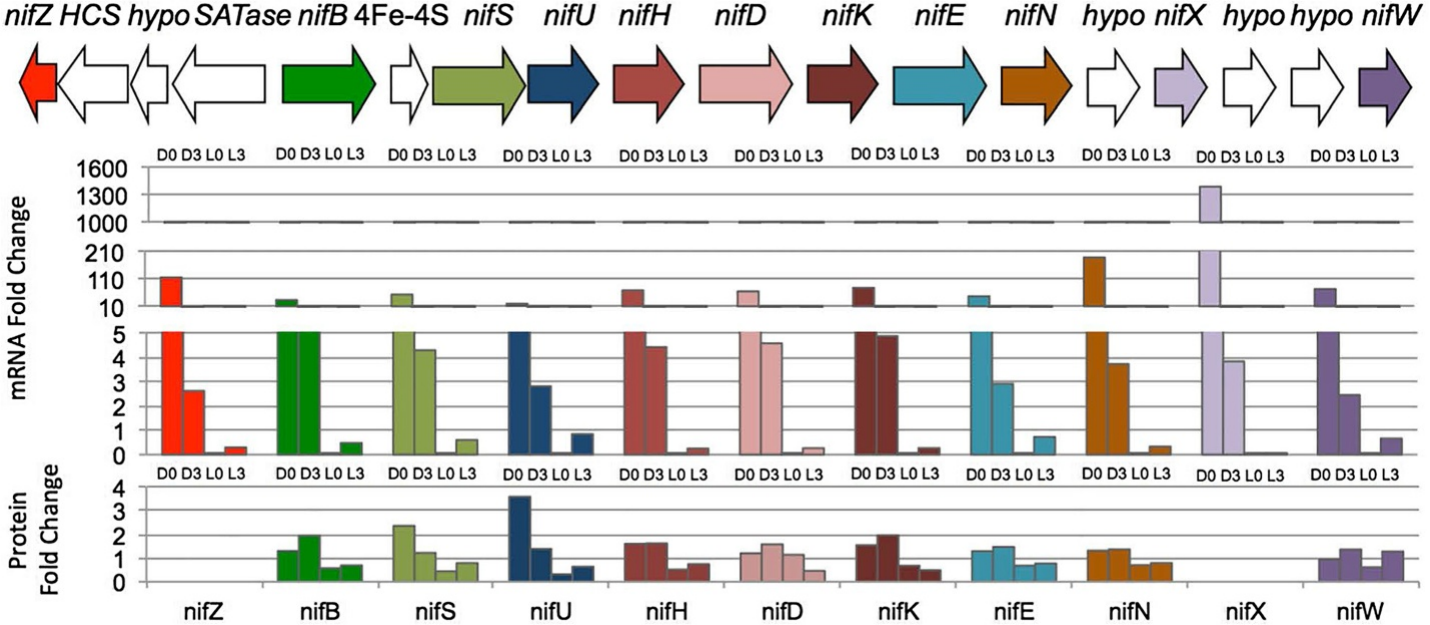
**Genes in the** ***nif*** **cassette in** ***Cyanothece*** **7822 display consistent expression in both mRNA and protein during the dark phase.** It is notable that NifD show anti-phase abundance maxima with the respectable transcripts. Peptides for the genes *nifZ* and *nifX* were not detected in the analysis. The x-axis represents the four comparisons: D0 vs. L3 (mid-day to sunset); D3 vs. D0 (mid-night to sunset); D3 vs. L0 (mid-night to sunrise); and, L3 vs. L0 (sunrise to early morning).

The relationship between transcription and translation was one of the main objectives of this study and we again concentrated on the key metabolic categories. The nitrogenase gene cluster represents an important standard for such comparisons and is shown in Figure 6. These genes are all located in a large cluster and appear to be coordinately regulated at the transcriptional level, primarily in the dark. We were able to detect all of the proteins at all time points, except for *nifZ* and *nifX*. For this group of genes, there was a great deal of correspondence between the timing of transcription and the appearance of the protein. The only one that was not in phase was nifD where the peak protein abundance lagged that of the mRNA. The protein abundance in the late light is high and quite similar to the mRNA pattern, thus establishing the nitrogenase cluster as an example of in-phase transcription/translation. The intensity of expression changes was substantial, with fold changes ranging from 10 to >1000 at the onset of the dark period. This emphasizes that the late light/early dark phase is the critical period for nitrogen fixation in *Cyanothece* 7822.

In regard to proteins involved in photosynthesis, not surprisingly most of photosystem I (PSI) and photosystem II (PSII) were higher in the light, whereas the cytb6/f complex and the ATPase synthase were fairly constant across the light periods. The photosynthetic antennae proteins that form the phycobilisomes were mostly light upregulated, except for the linker proteins that were somewhat higher in the dark. However, there were two interesting features that are also found in *Cyanothece* 51142: The PsaAB proteins were higher in the dark and one of the PsbA (D1) proteins (*psbA4*, Cyan7822_3753) was synthesized explicitly in the dark (some 2.5X higher at L0 (D12) than at the other time points). All sequenced cyanobacteria have multiple copies of *psbA* and some of these copies have mutations that likely render D1 incapable of binding the Mn_4_CaO_5_ metallocluster; thus, PSII complexes incorporating this altered DI would be incapable of O_2_ evolution [25]. We have noted that alternate copies of D1 are used by cyanobacteria under different environmental conditions, including high light, anoxic conditions or N_2_ fixation [26, 27]. We have postulated that under N_2_-fixing conditions, the expression of such a modified D1 will permit a complete PSII complex to form, but one that cannot evolve O_2_. The amount of PsbA4 in the dark indicated that this is an important feature in *Cyanothece* 7822.

**Figure 7:**
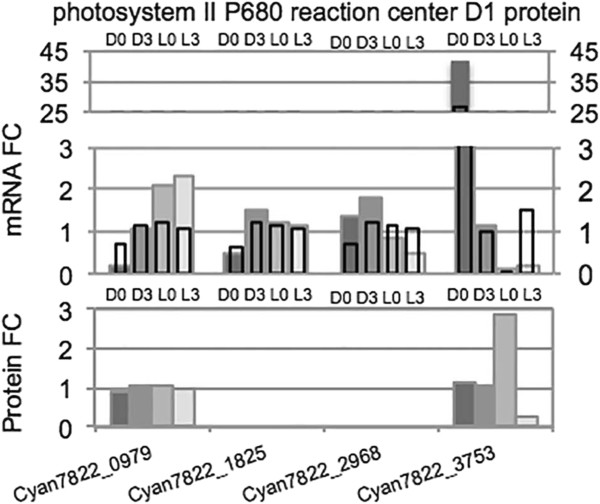
**Expression levels for the** ***psbA*** **genes in** ***Cyanothece*** **7822. Solid bars represent RNA-Seq and protein data and empty bars represent microarray data.** Due to their near identical sequences, D1 proteins *psbA1* (*Cyan7822_0979*), *psbA2* (*Cyan7822_1825*) and *psbA3* (*Cyan7822_2968*) were quantitatively indistinguishable at the protein level. Microarray data points consisted of fold changes at D0vsL4 (instead of L3), D4vsD0 (instead of D3vsD0), L0vsD4, and L4vsL0.

The *Cyanothece* 7822 genome contains 4 copies of the genes encoding the reaction center protein PsbA (also called D1). Three of these genes are virtually identical in sequence (*psbA1* (*Cyan7822_0979*), *psbA2* (*Cyan7822_1825*) and *psbA3* (*Cyan7822_2968*)) and cannot be differentiated at the protein level. Figure 7 shows that the RNA levels for these 3 proteins are fairly constant over the 4 time periods in both the RNA-Seq (filled bars) and microarray experiments (empty bars) and that the composite protein level was also quite constant. However, *psbA4* (*Cyan7822_3753)* mRNA was transcribed in the late light period and was high through the early dark. The protein was expressed in the late light and particularly strongly in the middle- to late-dark period. Thus, the D1 from *psbA4* became an important component during N_*2*_ fixation when O_2_ levels should be at their lowest.

#### Central metabolism

We collected a quite complete set of data for the enzymes in central metabolism and identified a number of different patterns of transcription and translation (Figure 5). In glycolysis, it appeared clear that differential expression at the transcriptional level had little to do with the abundance of the proteins at various times throughout the diurnal cycle (compare the mRNA panels with the protein panels within each metabolic pathway). Differential transcription at D0 vs L3, D3 vs D0, L0 vs D3, and L3 vs L0 varied, ranging from modest changes in the mRNA (*pyk*) to extreme induction (*fbp, pfk,* and *gap*), whereas the observed protein abundance changed very little in most cases. The few exceptions were Fbp1 (Cyan7822_4171), Pgk (Cyan7822_1455), Gap2 (Cyan7822_4638) and Fba2 (Cyan7822_1659), as shown in Figure 5. For these genes, each of the mRNA transcripts and respective proteins increased and decreased more or less in concert with a slight lag for the increase in protein abundance. The discrepancy in the abundance changes of mRNA and protein suggests that post-translational regulatory mechanisms play an important role for central metabolism in *Cyanothece* 7822.

In the pentose phosphate pathway, the mRNA changes are far more striking than those of the proteins. This pathway is used in both the light and the dark and, although the transcripts of many proteins were most abundant in the late dark and early light, the protein levels remained high throughout much of the 24h period. Interestingly, transcription of the genes for proteins also used for CO_2_ fixation was highest in the mid to late dark, thus preparing the cells for the onset of light-driven photosynthesis. This included the Rubisco genes for which the mRNA levels increased over 10-fold in the late dark and the protein levels were enhanced by about 2-folld in the late dark and the early light. The genes for the TCA cycle were somewhat different, in that the main mRNA induction was typically in the daytime and the proteins seemed to be at slightly higher abundance in the light. Glycogen/starch metabolism is also represented by a number of different relationships where the proteins remained more stable than the mRNA levels would have indicated. The one instance where transcription and translation are in phase is the *cphA* gene that encodes the cyanophycin synthase. The expression patterns both are highest in the late light and progressively diminish during the dark into the early light. CphA is most needed during and after nitrogen fixation and so a high level at these times is appropriate.

### Protein expression across genomic elements

Another objective was to analyze gene expression across the multiple elements that comprise this relatively complex genome. The genome of *Cyanothece* 7822 is the largest of the *Cyanothece* strains sequenced to date and is comprised of 6.1 Mb circular chromosome, in addition to 6 extra-chromosomal elements, ranging in size from 879 Kb to 13 Kb. An overview of how protein expression occurs from each genomic element is presented in Table 1. Over the 24 h time course, 1036 proteins were expressed with high quantitative reproducibility (see Methods section) from genes located on the main chromosome representing 18% of the presently annotated genes. However, a total of 1748 proteins from the circular chromosome were identified, at varying levels of quantitative accuracy, which accounted for representation of 31% of the total genes. All of the other genomic elements demonstrated some protein expression and the largest linear plasmid had 53 expressed proteins or 9% of the total ORFs on this plasmid. This genomic element encodes a number of very large genes and we obtained protein, of a variety of different functions, from 4 of these genes. Large expressed proteins included: Cyan7822_5813 with 1380 aa, which was at much higher levels in the early light. This protein may be involved in non-ribosomal peptide synthesis and could be involved with cyanophycin metabolism. Cyan7822_5889 encoded a 1211 aa protein that may be involved with Acyl-CoA synthesis and expression was highest in the late dark-early light periods. Cyan7822_5891 was among the largest polypeptides with 11,342 aa and expression was relatively unchanged throughout the day. This protein has many possible functional domains, but the largest component had homology to outer membrane adhesion-like proteins. Cyan7822_6000 with 5,687 aa was expressed with a peak in the dark and had homology to Concanavalin A-like lectin or glucanase. This protein does have a potential signal sequence at the N-terminus and may be located in the cytoplasmic membrane, but the remainder of the protein does not have transmembrane segments. In addition, a protein encoded by the 43kb plasmid was also large and upregulated in the light (Cyan7822_6655 with 1847 aa). The large proteins encoded by the chromosome cover many different functions as shown in Additional file 7: Table S4.

**Table 1:** **Protein Expression in** ***Cyanothece*** **7822 during 12h, LD, N**_**2**_ **-fixing growth [rounded to the nearest half percent]**

Element	Proteins expressed	Total genes on element	% Genes expressed
chromosome NC_014501 6.1 MB	1036	5663	18
plasmid NC_014533 879 kb	53	595	9
plasmid NC_014534 473 kb	7	422	1.5
plasmid NC_014502 291 kb	1	280	0.5
plasmid NC_014503 47 kb	3	32	9
plasmid NC_014504 43 kb	1	36	3
plasmid NC_014535 13 kb	1	13	7.5

### Anti-sense and CRISPR expression

The RNA-Seq experiment provided an opportunity to assess anti-sense RNA transcription in *Cyanothece* 7822. We know little about the overall importance of anti-sense RNA in cyanobacteria, but we can correlate global patterns in the light and dark and in regards to specific genomic elements. Anti-sense RNA was found throughout the genome, with sizes ranging from ~50 nt to over 500 nt. In addition, the anti-sense RNA density plot indicated that there were different levels of anti-sense throughout the light–dark cycle and that the amount of anti-sense RNA was lowest at D3. In analyzing the anti-sense as a function of genomic elements, the density was somewhat lower on plasmid 4, the 879 kb linear plasmid, and lowest on the 879 kb plasmid at D3. This relationship was verified by a more detailed statistical analysis as shown in Table 2. The main chromosome had a greater percentage of anti-sense RNAs than expected, whereas the 879 kb plasmid had many fewer anti-sense RNAs than expected, based on the total number of anti-sense RNAs detected. Although we have no direct indication that antisense-transcription regulates any specific cellular processes, the expression of antisense mRNA is not equal at the four time points measured. Such differences suggest that the antisense RNAs are modulated in response to the light–dark cycle and this may indicate a role in diurnal regulation.

**Table 2:** **Antisense RNA vs Genome**

		chromosome NC_014501	NC_014533	NC_014534	NC_014502	NC_014503	NC_014504	NC_014535	Total	Plasmid count	%
	All genes	5330	558	362	246	29	31	10	6566	1236	18.8
**D0**	Count	978	66	48	47	4	9	2	1155	177	15.3
	Expected	937.2	98	64.1	43.3	5.1	5.4	1.8			
	p-value	6.40E-04*	1.6E-04*	0.026*	0.51	.591	0.097	0.33			
**D3**	Count	921	55	58	42	2	7	3	1088	167	15.3
	Expected	883	92.5	60.2	40.7	4.7	5.1	1.7			
	p-value	9.20E-04*	5.80E-06*	0.74	0.82	0.18	0.37	0.26			
**L0**	Count	994	63	66	41	2	5	2	1173	179	15.3
	Expected	951.7	99.7	64.9	44.1	5.2	5.5	1.8			
	p-value	4.30E-04*	2.30E-05*	0.88	0.6	0.12	0.8	0.9			
**L3**	Count	1069	71	63	36	3	7	2	1252	183	14.6
	Expected	1016.4	105.9	69.4	46.8	5.5	6,1	1.9			
	p-value	1.30E-05*	7.20E-05*	0.39	0.068	0.21	0.67	0.39			

* Indicates significance with p < 0.25.

One of the most interesting outcomes was the level of expression of specific genes found on this large linear plasmid 4 (Figure 4). Of the 595 genes, 15 or 2.5%, are related to CRISPR activities in the cell. CRISPR genes have been found in many bacteria and provide them with adaptive sequence-specific nucleic acid restriction mechanisms that act as a defense against invading mobile genetic elements such as bacteriophages, invading plasmids, and transposons [28–33]. The presence of CRIPSR genes in *Cyanothece* is not unique to *Cyanothece* 7822. In fact, all sequenced *Cyanothece* strains contain at least 10 annotated CRISPR-related genes (see Additional file 8: Table S5). For example, *Cyanothece* 51142 has 12 annotated CRISPR genes on its genome, all of which are located on the main circular chromosome (NC_010546) with none on the linear chromosome or the 4 plasmids. In similar fashion, *Cyanothece* sp. ATCC 51472 and *Cyanothece* sp. PCC 8801 also contain all of their 12 or 11 CRISPR genes, respectively, on their main chromosomes. Other *Cyanothece* strains, such as *Cyanothece* sp. PCC 8802, 7425, 7425, and 7822 contain genes of this nature on both the main chromosome as well on their extra-chromosomal plasmids. *Cyanothece* 7822 is most interesting because there is a total of 23 genes of CRISPR related functions, but only one gene is found on the main chromosome (NC_014501), whereas 15 are found on the large linear plasmid (NC_014533) and 7 on the second largest linear plasmid (NC_014534).

## Discussion

### Comparison between Cyanothece strains

A number of proteomic studies have now been performed on a series of *Cyanothece* strains, including the best studied, *Cyanothece* 51142. An intensive proteomic study of *Cyanothece* 51142 was performed [10], followed by a more detailed analysis of those proteins differentially expressed on cells grown under 15 different conditions [12]. This later study identified some 250 proteins (5% of the predicted ORFs) that were differentially expressed. Their results for cells grown under nitrogen-fixing conditions were similar to what was found here for *Cyanothece* 7822. One significant difference was that *Cyanothece* 51142 proteins from genes encoding nitrogen-fixing and photosynthesis proteins were more highly synchronized to either the dark or light, respectively. On the other hand, the results for proteins involved in central metabolism were similar in both organisms.

A number of other cyanobacteria have been analyzed using RNA Seq including *Synechococcus* sp. PCC 7002 [34], *Anabaena* sp. PCC 7120 [35], *Microcysits aeruginosa* LE-3 [36] and *Prochlorococcus* MED4 [24]. The *Prochlorococcus* study was fundamentally the most similar to the current study, as it also analyzed proteomic data and obtained samples throughout a light–dark cycle. *Prochlorococcus* is the ocean’s most abundant primary producer and must always live according to the natural diel cycle. Analysis of transcriptional patterns demonstrated a substantial amount of light (or dark) induced mRNA, but the protein levels sometimes differed sharply from that of the mRNA. Waldbauer et al. [24] analyzed time courses of paired mRNA-protein abundances for 312 proteins every 2 h that represents about 16% of the 1955 ORFs in the genome. This is similar to the overall percentage from *Cyanothece* 7822 that we studied. In the case of both *Cyanothece* strains analyzed in detail, the closest convergence for mRNA and protein were for the nitrogenase cluster in the night—this seems to be critical, since the Nif complex is readily degraded by O_2_ that is most prevalent in the light.

### Future potential for study in Cyanothece 7822

**Figure 8:**
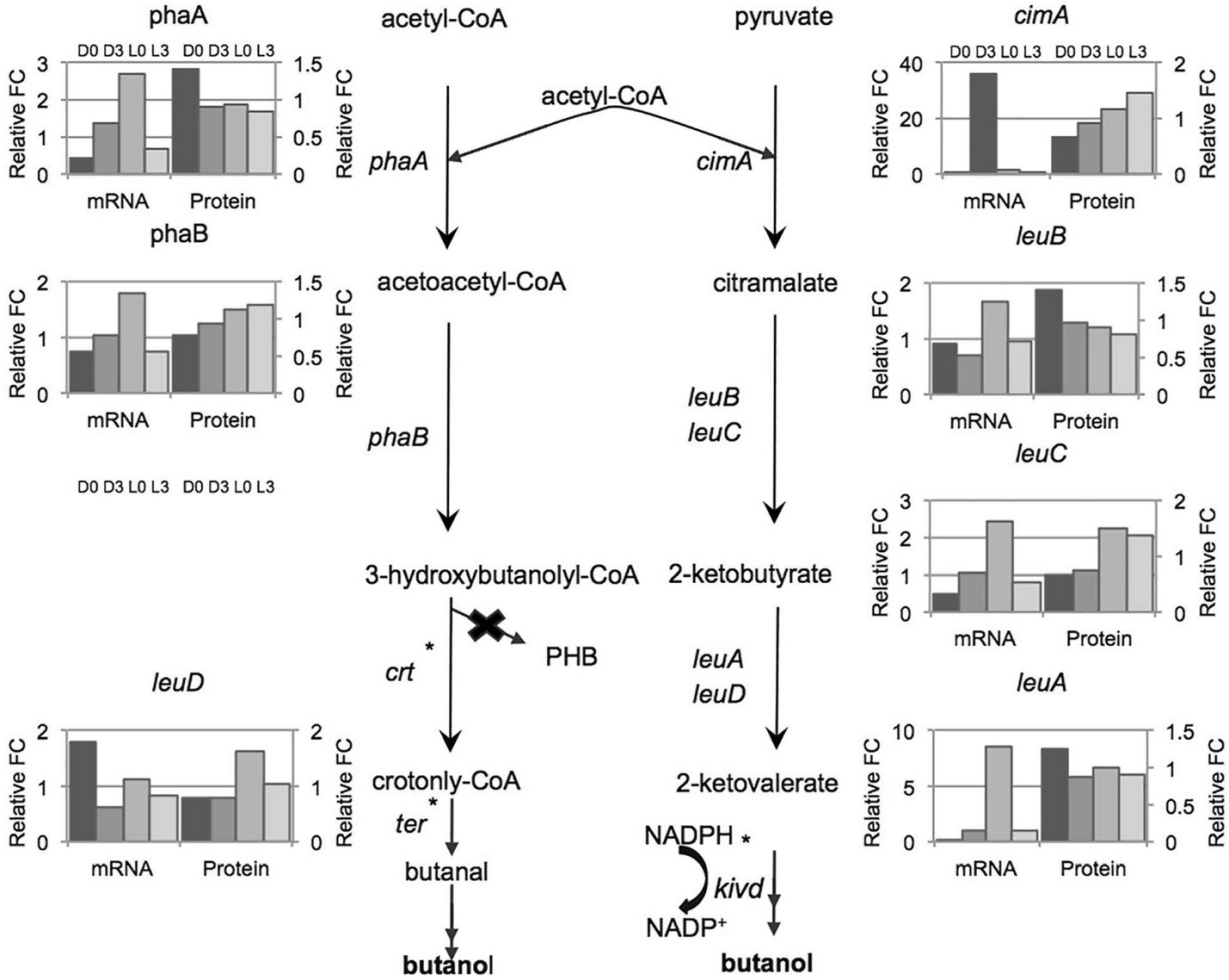
**The pathway required for generating the intermediates required for butanol production in** ***Cyanothece*** **7822 is shown, along with the expression values for mRNA and protein.** The x-axis represents the four comparisons: D0 vs. L3 (mid-day to sunset); D3 vs. D0 (mid-night to sunset); D3 vs. L0 (mid-night to sunrise); and, L3 vs. L0 (sunrise to early morning).

Another benefit of having paired mRNA and protein data is the ability to understand better the relationship of transcription and translation of metabolic pathways that generate intermediates for various targeted products. For example, *Cyanothece* 7822 has potential to be engineered for butanol production. *Cyanothece* 7822 possesses at least two pathways for butanol production (Figure 8), via isoleucine biosynthesis and via the crotonyl-CoA pathway. Isoleucine synthesis in *Cyanothece* 7822 is similar to that in *Cyanothece* 51142 and occurs through an alternate citramalate pathway similar to anaerobic bacteria and archaea [37]. Citramalate is generated through a condensation reaction of pyruvate and acetyl-CoA catalyzed by CimA then to 2-ketobutyrate and eventually isoleucine. The catalytic actions of the native LeuABCD that convert 2-ketobutyrate to 2-ketovalerate, along with the additional actions of a heterologous 2-ketoacid-decarboxylase (KdcA) and a dehydrogenation reaction, would theoretically allow butanol production. Generating butanol via the crotonyl-CoA pathway would require inhibition of PHB synthesis along with the introduction of two heterologous genes, 3-hydroxybutyryl dehydrogenase (*crt*) and trans-2-enoyl-CoA reductase (*ter*). Since *Cyanothece* is missing these last two genes in the biosynthesis pathway, as well as a 2-keto decarboxylase, it is proposed that the introduction of these 3 genes into *Cyanothece* 7822 would result in the phototrophic production of butanol. This is similar to genetic manipulations in *Synechococcus elongatus* PCC 7942 that led to the production of butanol [38] and isobutyraldehyde [39]. In both these pathways, a variety of comparative patterns can be seen, including in-phase and anti-phase. These results indicate that many different relationships occur between sense and antisense transcription and translation in *Cyanothece* 7822 and that targeting this strain to produce a specific metabolite will require in depth analysis of expression patterns, paying careful attention to the interplay between sense and antisense transcription and their relationship to translation levels.

While techniques to genetically modify *Cyanothece* 7822 to carry out these modifications exist, advancements in commandeering the *cas9* CRISPR enzyme for genome editing in bacteria could provide an alternative, and possibly, more efficient method [40, 41]. *Cyanothece* 7822 has a cluster of CRISPR genes on the extra-chromosomal plasmids. These genes display high levels of transcription, yet of the 23 genes annotated as being involved in CRISPR activities that are transcriptionally active, only one was observed in the protein population. While it is unclear what role these active genes are playing in the cell, their activity suggests that the introduction of *cas9*-based editing tools may be a viable new option that should be pursued further. In addition, it is notable that CRISPR-like genes are located on the ends of the large plasmid, a fact that allows the speculation that the pressure for maintenance of these extra-chromosomal plasmids is connected to their presumptive anti-viral activity. The reason for the complexity of the *Cyanothece* genomes [3] is unknown, but the possibility of horizontal gene transfer of DNA elements, followed by stabilization, is one possibility. The importance of specific gene functions on these introduced elements can be the reason that they have been preserved. We need to study gene expression in *Cyanothece* 7822 under many different conditions in order to test this general hypothesis and know exactly how these extra chromosomal elements are providing a benefit for the cell and it will be interesting to see if cyanophage that infect any of the *Cyanothece* strains can be identified in the future.

## Conclusions

An understanding of bacterial complexity requires the analysis of the full complement of proteins and the way they are regulated. We have begun this process in *Cyanothece* 7822 by analyzing transcription and translation across a 24 h diurnal period under nitrogen-fixing conditions. Overall, the results indicate that: (1) RNA Seq and microarray data provide similar information on mRNA transcription, although RNA Seq provides more coverage and higher sensitivity; (2) Although we can obtain complete coverage at the mRNA level, the number of proteins identified represents about 25% of all possible gene products; (3) Differential expression at the mRNA and protein level can follow the same general pattern or can be quite different. Strongly up- or down-regulated genes at the transcriptional level are no guarantee that the proteins will behave similarly; (4) Nonetheless, many proteins involved in key metabolic functions demonstrated expression to a greater extent in either the light or the dark, depending upon the need for that function. The dynamic range of the protein levels under these conditions is typically far less than that shown at the messenger RNA level, implying that statements relating to absolute levels of induction/repression at the transcriptional level may not be important at the protein level.

## Methods

### Cell cultivation

*Cyanothece* 7822 was grown in modified BG-11 medium [16] with the nitrate removed. For the RNA-seq analysis, cultures were grown in 100 ml volumes in 250 ml Erlenmeyer flasks at 30°C with shaking at 125 rpm and 30–40 μmol photons m^-2^ s^-1^ of light from cool white fluorescent bulbs; or 2. For the microarray analysis, cultures were grown in airlift bioreactors (6-liter BioFlo 3000; New Brunswick Scientific, Edison, NJ) illuminated by two light-emitting-diode panels using alternating arrays of orange (640 nm) and red (680 nm) light, providing an intensity of ∼ 100 μmol photons m^-2^ s^-1^ at 30°C. Experimental conditions consisted of 12-h light-12-h dark cycles for 3 to 4 days. Stock cultures grown in continuous light were used to inoculate experimental flasks at a density of approximately 1 × 10^6^ cells ml^-1^. For all RNA and protein collection cellular material was cooled to ~4°C with a refrigerated centrifuge and the material was stored on ice.

### Extraction and processing of mRNA

For RNA-Seq analysis, cellular material from each time point was cooled on ice to limit metabolic changes, kept at 4°C, and transported to the Environmental Molecular Sciences Laboratory at Pacific Northwest National Labs in Richland, WA. The RNA was extracted using Invitrogen TRIzol® Reagent (cat#15596018), followed by genomic DNA removal and cleaning using the Qiagen RNase-Free DNase Set kit (cat#79254) and the Qiagen Mini RNeasy™ kit (cat#74104). The Agilent 2100 Bioanalyzer was used to assess the integrity of the RNA samples. Only RNA samples having RNA Integrity Number ≥ 8 were used for RNA Seq. Deep RNA sequencing was performed on the 5500xl SOLiD™ sequencing platform (Life Technologies). Applied Biosystems SOLiD™ Total RNA-Seq kit (cat# 4445374) was used to generate the template cDNA library following the manufacturer recommended protocol. Briefly, the mRNA was fragmented using chemical hydrolysis followed by ligation with strand-specific adapters and reverse transcription to generate cDNA. Fragments of cDNA between 150 to 250 bp were subsequently isolated by electrophoresis in 6% urea-TBE acrylamide gel. The gel-isolated cDNA fragments went through 15 amplification cycles to produce enough templates for the SOLiD™ EZ Bead™ system (Life Technologies), which was used to perform emulsion clonal bead amplification to generate a template bead library for ligation-base sequencing using the SOLiD™ 5500xl instruments. Samples were analyzed at the Environmental Molecular Sciences Laboratory (EMSL) at Pacific Northwest National Laboratory (Richland, WA, USA). At times, cells were transported at 4°C and briefly thawed at room temperature prior to analysis.

For microarray analysis, RNA was extracted immediately after harvesting cells at each time point (D0, D4, L0 and L4) using Tri-Reagent® (Sigma-Aldrich cat#T9424) according to the manufacturer's protocol. Samples were resuspended in Tri-Reagent and washed with glass beads at temperature below 0°C followed by phase separation using 1-bromo-3-chloropropane. The time points were slightly different than in the RNA-Seq experiment, but we have shown that there is very little difference in expression levels between 3 and 4 h in either the light or dark in *Cyanothece* 7822. RNA was precipitated with isopropanol, and RNA clean-up kit-5 columns from Zymo Research Corporation (Orange, CA) were used to remove contamination. The hybridization control consisted of a mixed sample that contained equal amounts of RNA from each time point. Four biological and two technical replicates were included in analysis. For each microarray, 7 μg RNA was used (3.5 μg sample plus 3.5 μg control). Total RNA was labeled with either cyanine-5 or cyanine-3 by using a ULS RNA fluorescent-labeling kit from Kreatech Biotechnology (Amsterdam, The Netherlands) according to the manufacturer's protocol. The labeled material was passed through Zymo RNA clean-up kit-5 columns (Zymo Research Corporation, Orange, CA) to remove unincorporated label and eluted in 15 to 20 μl of RNase-free water. The concentration of labeled total RNA and label incorporation was determined on a Nanodrop-1000 spectrophotometer (Wilmington, DE). All of the labeling and post labeling procedures were conducted in an ozone-free enclosure to ensure the integrity of the label. The microarray platform consisted of 6538 ORFs based upon the *Cyanothece* genome sequence. The 60-mers appropriate for each gene were determined by MoGene and hybridization; scanning, and initial data analysis was conducted at MoGene (St. Louis, MO).

### Generation of RNA Seq data-files

The 50-base short read sequences produced by the 5500XL SOLiD™ sequencers were mapped to the *Cyanothece* 7822 reference genome (http://www.ncbi.nlm.nih.gov/genome/) using the default settings in the Life Technologies software provided for the SOLiD sequencers (LifeScope ver. 2.5 for the SOLiD 5500xl). LifeScope Whole Transcriptome analysis generated the following files: 1) a BAM file containing the sequence of every mapped read and its mapped location; 2) pairs of *.wig files (one for each strand on the chromosome) with the mapped counts at each base position; and 3) a gene counts file, with base counts summed to a single value across the entire gene length and RPKM values given for each gene.

### Semiquantitative RT-PCR

RNA was treated with DNase I (Invitrogen, Carlsbad, CA) for 1 h at 37°C and followed by DNase I treatment for 30 minutes at 37°C. Reverse transcription (RT) was performed by using Superscript II (Invitrogen, Carlsbad, CA) with random primers following the manufacturer's instructions. PCR was carried out at 94°C for 1 min, 30 cycles of 94°C for 30 s and 53°C for 30 s, and 72°C for 30 s to amplify regions of the genes *hoxH, hupL nifD, psbA*, and *rpnB*. PCR was carried out to amplify regions of the genes for *rnpB* (22 cycles), *hoxH* (32 cycles), *hupL* (28 cycles), *nifD* (32 cycles), and *psbA* (30 cycles). The *rpnB* transcript abundance was used as a control, since results from the microarray analysis indicated that the transcript level for this gene was unchanged under these growth conditions across each time point. The primers for each transcript were as follows: *rnpBF 5’-CGTGAGGATAGTGCCACAGA-3’, rnpBR5’- AAACGGGACCGGTAAAAGAC-3’, hoxHF 5’-GCTGAAGCCGGAATTAACAA-3’, hoxHR 5’-ATTTGTAGCGGCATTTGTCC-3’, hupLF 5’-AACGGTAAACCGATCAAACG-3’, hupLR 5’-CGGATGGGTCTTGATATTGG-3’, nifDF 5’-ATTTCCAAGAACGCGACATC-3’, nifDR 5’-TCACGAACAGCATCGTTAGC-3’, psbAF 5’-CCCACCCTTCTGACAGCTAC-3’, psbAR 5’-CTAACTGGTAAGGGCCACCA-3’.*

### Protein sample preparation

Cell suspensions were centrifuged for 30 min at 1000 × g at 4°C, reduced to 0.5 mL volume, and then washed at 4°C with 2 mL of NH_4_HCO_3_, pH 7.8, three times. To the final pelleted cells, 0.5 mL of lysis buffer (8 M Urea in NH_4_HCO_3_, pH 7.8) was added and they were homogenized for 5 min using a Qiagen TissueRuptor followed by a BCA assay (Pierce) to determine protein concentration. Protein samples were incubated in 8 M Urea, 5 mM dithiothreitol at 56°C for 45 min with shaking at 800 rpm in Thermomixer R (Eppendorf). Alkylation was performed with 20 mM iodoacetamide at 37°C in dark with shaking (800 rpm in Thermomixer) followed by and 8 fold dilution with nanopure water containing 1 mM CaCl_2_. Tryptic digest with Sequencing Grade Modified Trypsin (Promega) was performed at 1:50 enzyme‒to‒substrate ratio for 4 h at 37°C. The digested samples were then acidified with 10% trifluoroacetic acid to ~ pH 3 and 5% acetonitrile was added to the digested samples prior to desalting. SPE C-18 columns (SUPELCO Discovery) were used for clean-up of the resultant peptide mixture, and samples were concentrated down using the SpeedVac SC250 Express (ThermoSavant) followed by BCA assay to determine final peptide concentration.

### iTRAQ labeling and HPLC fractionation

Isobaric labeling of captured peptides to achieve relative quantification using four-plex iTRAQ™ reagents was performed according to the manufacturer’s instructions (AB Sciex, Foster City, CA). For iTRAQ™ labeling, 30 μg of peptide for each time point was resuspended in 10.0 μL of dissolution Buffer (500 mM triethylammonium bicarbonate) mixed with the individual iTRAQ™ reagent and incubated at RT for 1 h. The reaction was stopped and unreacted iTRAQ reagents were hydrolyzed by adding 150 μL of H_2_O and incubation at RT for 30 min. Contents of each iTRAQ reagent-labeled sample was then pooled, followed by concentration down to 100 μL prior to HPLC fractionation.

Labeled peptide samples were separated at flow rate at 0.5 mL/min on a reverse phase Waters XBridge C18 column (250 mm × 4.6 mm column containing 5-μm particles, and a 4.6 mm × 20 mm guard column) using an Agilent 1200 HPLC System equipped with a quaternary pump, degasser, diode array detector, Peltier-cooled auto-sampler and fraction collector (both set at 4°C). 120 μg of labeled tryptic peptides was suspended in buffer A (10 mM triethylammonium bicarbonate, pH 7.5) and loaded onto the column. After the sample loading, the C18 column was washed for 35 min with solvent A, before applying the LC gradient. The LC gradient started with a linear increase of solvent A to 10% B (10 mM triethylammonium bicarbonate, pH 7.5, 90% acetonitrile) for 10 min, then linearly increased to 20% B at 15 min, 30 min to 30 % B, 15 min to 35% B, 10 min to 45% B and another 10 min to 100% solvent B. Using an automated fraction collector, 96 fractions were collected for each sample, lyophilized and reconstituted into 12 fractions prior to LC-MS/MS analysis. These 12 final fractions were concatenated by combining each 8 fractions that were 12 fractions apart.

### LC-MS/MS analysis

All iTRAQ™-labeled fractions were analyzed by LC–MS/MS. Each sample was loaded onto a homemade 65 cm × 75 mm i.d. reversed-phase capillary column using 3-mm C18 particles (Phenomenex, Torrance, CA, USA). The HPLC system consisted of a custom configuration of 100-ml Isco Model 100DM syringe pumps (Isco, Lincoln, NE, USA), two-position Valco valves (Valco Instruments Co., Houston, TX, USA), and a PAL autosampler (Leap Technologies, Carrboro, NC, USA) that allowed fully automated sample analysis across four HPLC columns [42]. The system was operated at a constant pressure of 10,000 psi over 3 h with an exponential gradient starting with 100% of mobile phase A (0.1% (v/v) formic acid in water) to 60% (v/v) of mobile phase B (0.1% (v/v) formic acid in acetonitrile). MS analysis was performed on a Thermo Scientific LTQ-Orbitrap Velos mass spectrometer (Thermo Scientific, San Jose, CA, USA) coupled with an electrospray ionization interface using home-made 150-mm o.d. × 20-mm i.d. chemically etched electrospray emitters [43]. Full MS spectra were recorded at resolution of 100 K (m/z 400) over the range of m/z 400–2000 with an automated gain control (AGC) value of 1 × 10^6^. MS/MS was performed in the data-dependent mode with an AGC target value of 3 × 10^4^. The most abundant 10 parent ions were selected for MS/MS using high-energy collision dissociation with a normalized collision energy setting of 45. Precursor ion activation was performed with an isolation width of 2 Da, a minimal intensity of 500 counts, and an activation time of 10 ms.

### Data analysis

LC–MS/MS raw data were converted into data files using Bioworks Cluster 3.2 (Thermo Fisher Scientific, Cambridge, MA, USA), and MSGF+ algorithm [44] was used to search MS/MS spectra against the *Cyanothece* 7822 sequence (NCBI 2011-02-28). The key search parameters used were 20 ppm tolerance for precursor ion masses, partial tryptic search, dynamic oxidation of methionine (15.9949 Da), static IAA alkylation on cysteine (57.0215 Da), and static iTRAQ modification of lysine and N-termini (+144.1021 Da). The decoy data base searching methodology [45, 46] was used to control the false discovery rate at the unique peptide level to ~0.1% [47]. For quantification purposes, peptide reporter ion intensities were captured across all channels and compared by calculating the summed peptide intensity protein ratio between L3, D0, and D3 versus L0. Protein values with a correlation of >0.5 between biological replicates were retained. Certain proteins, such as RbcLS, with a correlation of 0.46 were included in our analysis. These highly abundant proteins fluctuated among our biological replicates, but always had the same periodicity and were important for the overall analysis. Protein values were then central tendency normalized using the program DAnTE [48] for direct comparison against mRNA values.

## Electronic supplementary material

Additional file 1: Table S1: mRNA Summary. (XLSX 464 KB)

Additional file 2: Table S2: Functional mRNA Expression. (XLSX 403 KB)

Additional file 3: Figure S1: Four scatter plots to illustrate the correlation between the RNA Seq method (voom, RPKM) and the microarray method in terms of log2 fold changes. A) Illustrates the correlation of common significant differentially expressed genes (at FDR < 0.05) between the RNA Seq method (RPKM) and the microarray method for D0 vs L0 comparison on x-axis and y-axis respectively. The Spearman’s correlation coefficient for this comparison is 0.846. B) Illustrates the correlation of the common significant differentially expressed genes (at FDR < 0.05) between the RNA Seq method (voom) and the microarray method for a D0 vs L0 comparison on the x-axis and y-axis, respectively. The Spearman’s correlation coefficient for this comparison is 0.877. C) llustrates the correlation of all genes between the RNA Seq method (RPKM) and the microarray method for the D0 v L0 comparison on the x-axis and y-axis, respectively. The Spearman’s correlation coefficient for this comparison is 0.667 D) Illustrates the correlation of all genes between the RNA-Seq method (voom) and the microarray method for the D0 vs. L0 comparison on the x-axis and the y-axis, respectively. (TIFF 15 MB)

Additional file 4: Figure S2: Comparison heat map showing up (red) and down (green) regulation of the major metabolic genes in *Cyanothece* 7822 for the microarray platform and the RNA-seq method. (TIFF 4 MB)

Additional file 5: Table S3: Total mRNA Expression. (XLSX 6 MB)

Additional file 6: Figure S3: Acrylamide gel patterns of key metabolic genes in *Cyanothece* 7822 critical for nitrogen fixation and photosynthesis measured by reverse transcriptase PCR . The results correlated very closely to that of the RNA Seq and microarray experiments. (TIFF 3 MB)

Additional file 7: Table S4: Protein. (XLSX 234 KB)

Additional file 8: Table S5: CRISPR. (XLSX 41 KB)

## Abbreviations

PHB: Polyhydroxybutyrate.
