# Supplementary figures and images for: Transcriptomic and proteomic dynamics in the metabolism of a diazotrophic cyanobacterium, Cyanothece sp. PCC 7822 during a diurnal light–dark cycle
Source: BMC Genomics. 2014 Dec 29;15(1):1185. doi: 10.1186/1471-2164-15-1185 (PMC4320622; doi:10.1186/1471-2164-15-1185)

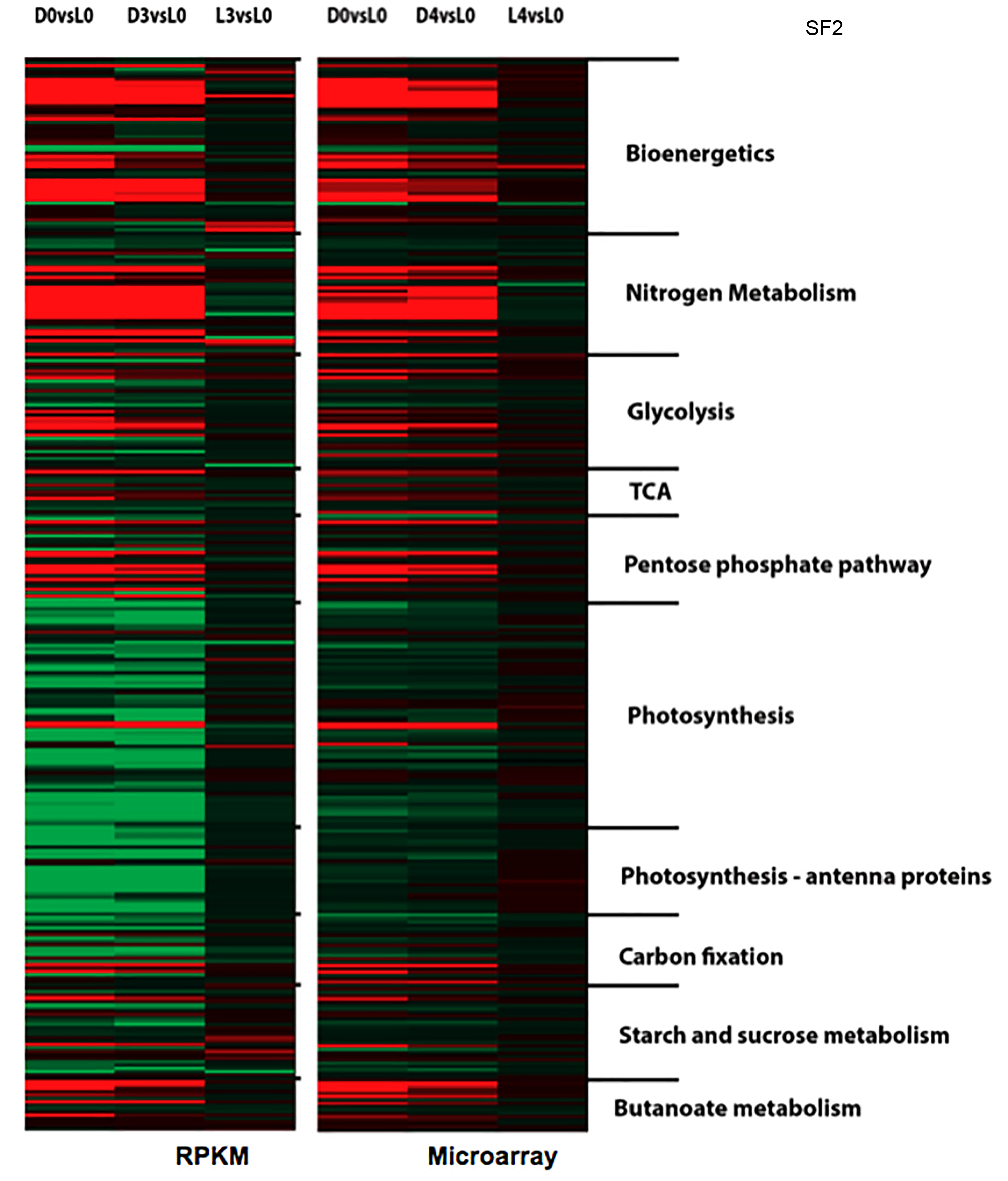

Supplement: Supplementary file 3 — Additional file 3: Figure S1: Four scatter plots to illustrate the correlation between the RNA Seq method (voom, RPKM) and the microarray method in terms of log2 fold changes. A) Illustrates the correlation of common significant differentially expressed genes (at FDR < 0.05) between the RNA Seq method (RPKM) and the microarray method for D0 vs L0 comparison on x-axis and y-axis respectively. The Spearman’s correlation coefficient for this comparison is 0.846. B) Illustrates the correlation of the common significant differentially expressed genes (at FDR < 0.05) between the RNA Seq method (voom) and the microarray method for a D0 vs L0 comparison on the x-axis and y-axis, respectively. The Spearman’s correlation coefficient for this comparison is 0.877. C) llustrates the correlation of all genes between the RNA Seq method (RPKM) and the microarray method for the D0 v L0 comparison on the x-axis and y-axis, respectively. The Spearman’s correlation coefficient for this comparison is 0.667 D) Illustrates the correlation of all genes between the RNA-Seq method (voom) and the microarray method for the D0 vs. L0 comparison on the x-axis and the y-axis, respectively. (TIFF 15 MB) [file 12864_2014_6950_MOESM3_ESM.tiff]

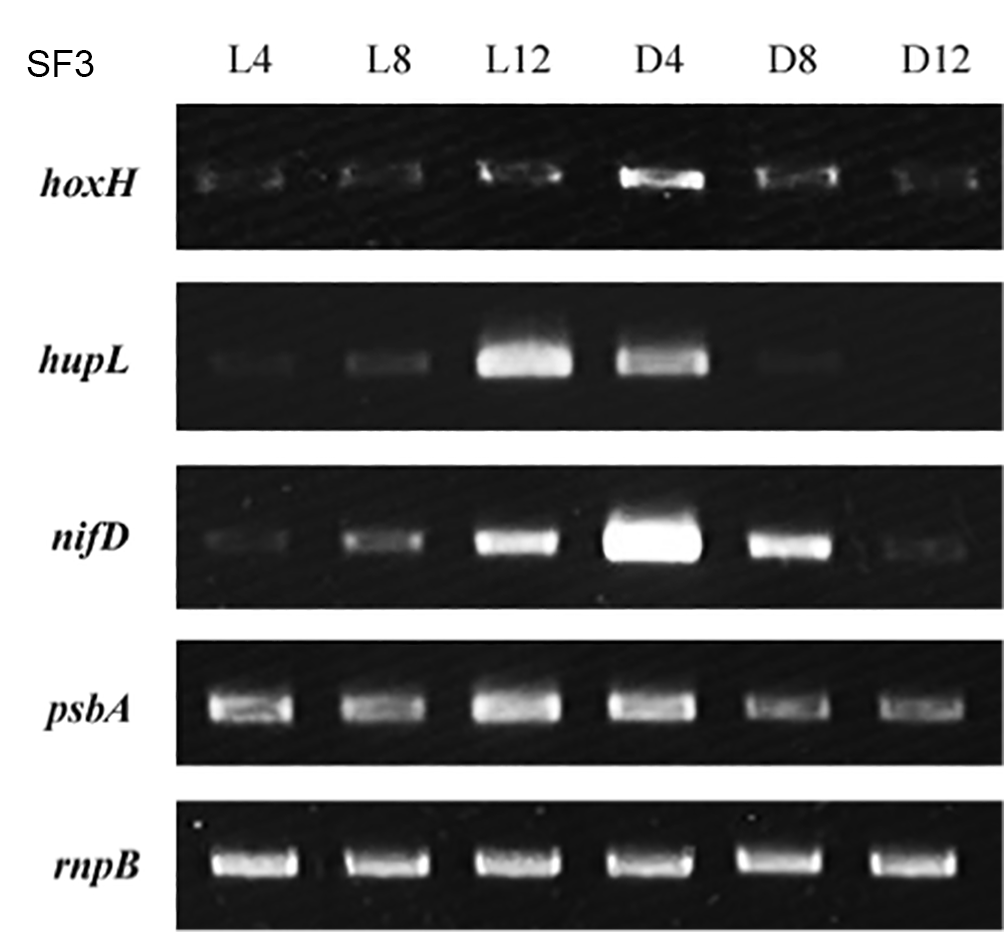

Supplement: Supplementary file 4 — Additional file 4: Figure S2: Comparison heat map showing up (red) and down (green) regulation of the major metabolic genes in Cyanothece 7822 for the microarray platform and the RNA-seq method. (TIFF 4 MB) [file 12864_2014_6950_MOESM4_ESM.tiff]

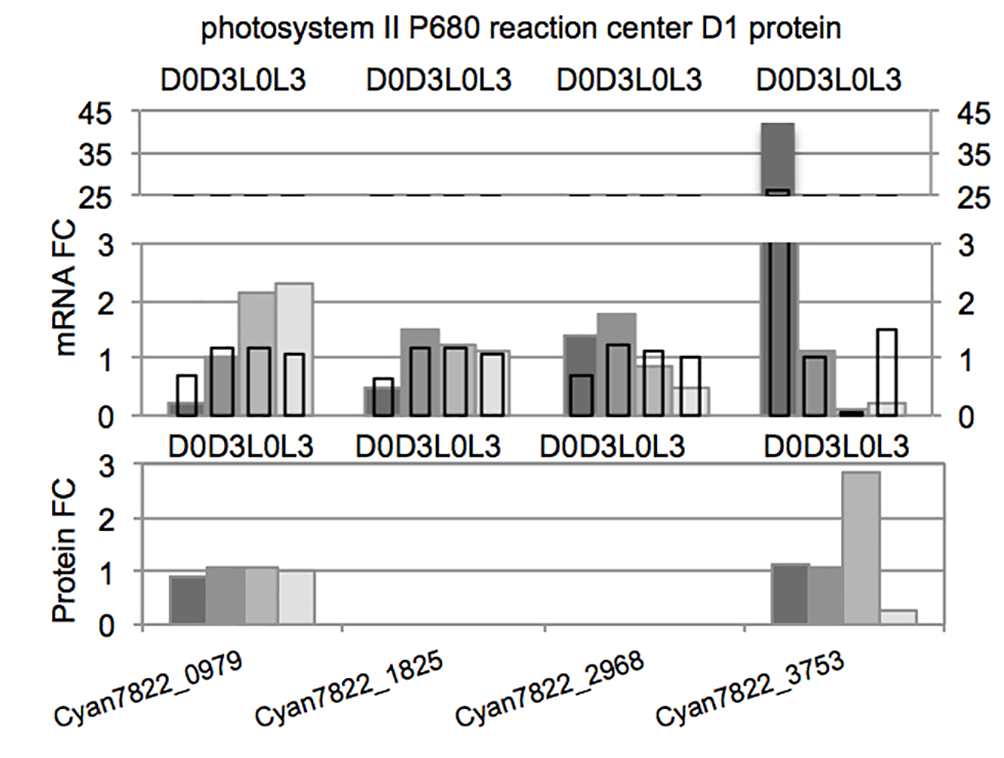

Supplement: Supplementary file 6 — Additional file 6: Figure S3: Acrylamide gel patterns of key metabolic genes in Cyanothece 7822 critical for nitrogen fixation and photosynthesis measured by reverse transcriptase PCR . The results correlated very closely to that of the RNA Seq and microarray experiments. (TIFF 3 MB) [file 12864_2014_6950_MOESM6_ESM.tiff]
